# Consecutive learning of opposing unimanual motor tasks using the right arm followed by the left arm causes intermanual interference

**DOI:** 10.1371/journal.pone.0176594

**Published:** 2017-05-01

**Authors:** Christian Stockinger, Benjamin Thürer, Thorsten Stein

**Affiliations:** 1 BioMotion Center, Institute of Sports and Sports Science, Karlsruhe Institute of Technology, Karlsruhe, Germany; 2 HEiKA–Heidelberg Karlsruhe Research Partnership, Heidelberg University, Karlsruhe Institute of Technology, Karlsruhe, Germany; 3 Neuromuscular Diagnostics, Department of Sport and Health Sciences, Technical University of Munich, Munich, Germany; Universitair Medisch Centrum Groningen, NETHERLANDS

## Abstract

Intermanual transfer (motor memory generalization across arms) and motor memory interference (impairment of retest performance in consecutive motor learning) are well-investigated motor learning phenomena. However, the interplay of these phenomena remains elusive, i.e., whether intermanual interference occurs when two unimanual tasks are consecutively learned using different arms. Here, we examine intermanual interference when subjects consecutively adapt their right and left arm movements to novel dynamics. We considered two force field tasks A and B which were of the same structure but mirrored orientation (B = -A). The first test group (ABA-group) consecutively learned task A using their right arm and task B using their left arm before being retested for task A with their right arm. Another test group (AAA-group) learned only task A in the same right-left-right arm schedule. Control subjects learned task A using their right arm without intermediate left arm learning. All groups were able to adapt their right arm movements to force field A and both test groups showed significant intermanual transfer of this initial learning to the contralateral left arm of 21.9% (ABA-group) and 27.6% (AAA-group). Consecutively, both test groups adapted their left arm movements to force field B (ABA-group) or force field A (AAA-group). For the ABA-group, left arm learning caused significant intermanual interference of the initially learned right arm task (68.3% performance decrease). The performance decrease of the AAA-group (10.2%) did not differ from controls (15.5%). These findings suggest that motor control and learning of right and left arm movements involve partly similar neural networks or underlie a vital interhemispheric connectivity. Moreover, our results suggest a preferred internal task representation in extrinsic Cartesian-based coordinates rather than in intrinsic joint-based coordinates because interference was absent when learning was performed in extrinsically equivalent fashion (AAA-group) but interference occurred when learning was performed in intrinsically equivalent fashion (ABA-group).

## Introduction

The human sensorimotor system is capable of learning a variety of motor tasks and generalizing previously learned motor tasks to different contexts. In particular, interlimb transfer (or interlimb generalization, cross-limb transfer, cross-education) refers to the generalization of motor memory from one limb to another. Existence of such interlimb transfer is well-known and corresponding investigations are relevant from a theoretical perspective–e.g., to investigate interhemispheric connectivity [1,2] or models of internal task representation [3]–but also for practical reasons like the design of effective interventions in rehabilitation or sports in terms of bilateral practice schedules [4–6].

To investigate motor learning and its transfer, arm movements are often considered. In this case, interlimb transfer refers to intermanual transfer (or intermanual generalization). Several studies found that adaptation of arm movements to novel dynamic conditions partly transfers to the contralateral arm [7–10]. In contrast to early findings, which detected such transfer only from the dominant to the non-dominant arm [7], more recent studies reported such transfer to occur in both directions [10,11]. These findings suggest a bidirectional interplay of the corresponding neural networks or the involvement of at least partly similar neural networks for motor control and learning of both arms. Intermanual transfer is often explained by the cross-activation model or the bilateral access model [2]. The cross-activation model states that unilateral practice causes bilateral neural adaptations in both the contralateral and the ipsilateral hemisphere. According to the bilateral access model, practice-dependent adaptations occur in neural regions which are accessible for both the trained and untrained arm. However, it is still under debate which neural networks are actually involved in the transfer of motor learning [1,2]. Moreover, the coordinate frame of intermanual transfer and, thus, the internal representation of the underlying task is under debate. Transfer might occur in an intrinsic joint-based reference frame [12–14], an extrinsic Cartesian-based reference frame [7,8,10], or in a combination of reference frames [3,15] with the weighting of this combination presumably being driven by the likelihood of each particular reference frame [16]. In arm movements which are performed near the body midline, intermanual transfer with respect to an intrinsic reference frame would result in motor behavior at the endpoint that is mirrored about the midsagittal plane. Intermanual transfer in extrinsic coordinates would result in identical motor behaviors at both arms, when considered from an external viewpoint [7]. It is assumed that ambiguous dynamics are learned in a preferred coordinate frame (e.g., extrinsic or intrinsic) but that the motor system can sculpt the particular coordinate representation in which the task is learned through experience [16].

A second important phenomenon in motor learning is that consecutively learning two different tasks A and B using the same arm can lead to motor memory interference, i.e., learning a second task B after initially learning task A affects retest performance of the previously learned task A (ABA-paradigm) [17–20]. In particular, when subjects learn to perform arm movements under altered dynamic conditions (task A), such interference occurs when the subsequently learned task B is of the same structure but reversed direction (task B = -A) [17,18,21–25]. Hereby, interference presumably arises because learning the tasks A and B involve the same neural networks as they share the same task structure and are learned and executed using the same arm. Thus, formation of task B motor memory might compete with the consolidation of the initially formed task A motor memory.

Yet, it is unknown whether motor memory interference also occurs when the two tasks are consecutively learned using different arms. Thus, the purpose of this study was to investigate the interplay of the two mentioned motor learning phenomena (*intermanual transfer* and *motor memory interference*), which will be denoted as *intermanual interference*. Therefore, we examined whether motor memory interference occurs across arms when subjects consecutively adapt their right and left arm movements to novel dynamic conditions. For this purpose, we considered two unimanual motor tasks A and B, where task B is of the same structure as task A but of mirrored orientation with respect to the body midline (i.e., B = -A). If the preferred reference frame of the motor task is an intrinsic, joint-based reference frame, the learned task A should correspond at the contralateral arm to a mirrored motor behavior according to task B. Contrary, if the motor task is preferentially represented in an extrinsic coordinate frame, the learned task A should also correspond to task A on the contralateral arm. Therefore, depending on the internal representation of the task, consecutive task learning in an AAA-schedule or an ABA-schedule should either result in motor memory interference or facilitation of motor learning. Based on previous findings of our laboratory [10], we hypothesized that intermanual transfer occurs in extrinsic coordinates and, thus, that consecutive unimanual right-left-right arm learning in an ABA-schedule leads to intermanual interference whereas consecutive unimanual learning in an AAA-schedule leads to motor performance facilitation at retest. Moreover, we assumed that repeated learning of task A and task B using the right and left arm, respectively, yields to a recognition of the alternating task patterns, thus, changing subjects’ initially preferred extrinsic task representation.

## Materials and methods

### Participants

A total of 36 healthy human subjects (18–30 years, 17 female) participated in the study. All subjects gave written informed consent and the test protocol was reviewed and approved by the ethics committee of the Karlsruhe Institute of Technology. All subjects were right-handed (83±19% according to the 10-item version of the Edinburgh Handedness Inventory; [26]) and were naïve to the experimental procedure (apparatus, paradigm, and purpose of the study). They were asked not to consume any alcohol or drugs during the test day and instructed to sleep at least 6 h in the nights prior to the test session.

### Apparatus

**Fig 1:**
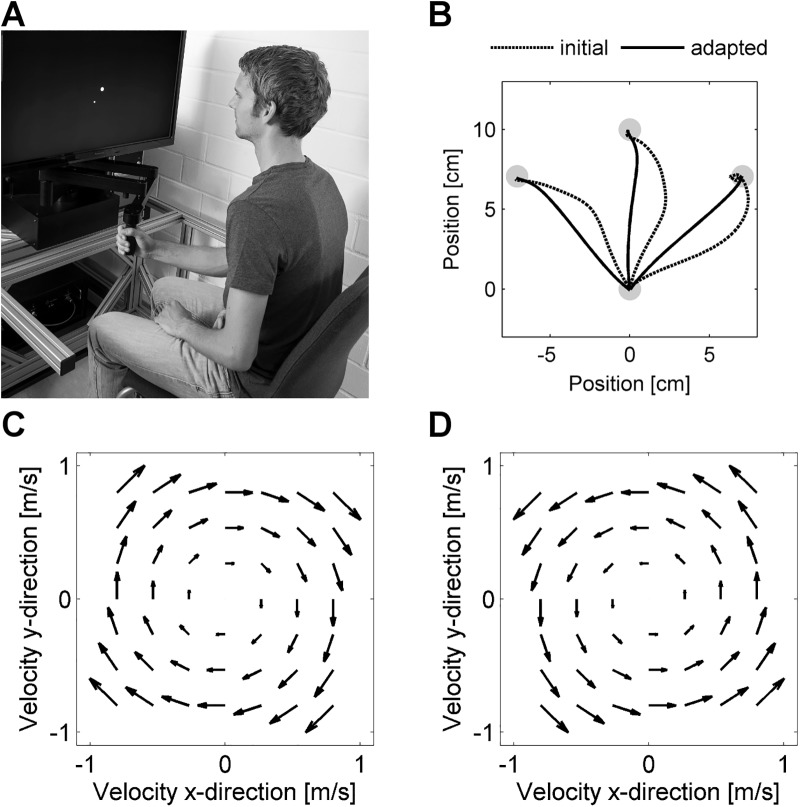
Motor adaptation task. (**A**) The subjects grasped the handle of a robotic device, performed goal-directed point-to-point reaching movements, and adapted their reaching movements to force field perturbations. (**B**) Typical mean movement paths (only outward directed shown) under force field conditions showing initially high deviations but straightened movement paths after adaptation. (**C**, **D**) The implemented force fields pushed the subjects’ arms perpendicular to the current movement direction depending on the movement speed. To create two force field tasks A and B, clockwise- and counterclockwise-directed force fields were implemented (the force field directions for tasks A and B were counterbalanced across subjects).

We used a robot-assisted experimental paradigm [12] which was designed similar to former investigations of our laboratory [10]. Subjects grasped the handle of a robotic device which could exert forces (Kinarm End-Point Lab, BKIN Technologies, Kingston, Canada; Fig 1A). Subjects’ arms were not supported and motion of the robot’s handle was restricted to the horizontal plane. Throughout the whole experiment, the subjects had clear view of their arm and they received full visual feedback of the targets as well as the cursor corresponding to the position of the handle on a vertical monitor which was positioned approximately at eye level. The robot was centrally positioned in front of the subjects such that the center position of the robot handle was located in the subjects’ midsagittal plane. Subjects sat on a chair such that they were able to comfortably grasp the handle with either hand and reach all target positions. Position and force at the handle were recorded at a sampling rate of 1000 Hz.

### Task

Subjects were asked to perform accurate goal-directed 2d point-to-point reaching movements using the robot handle (Fig 1B). Performing this motor task involved both elbow and shoulder motions. Starting from a center point, subjects had to reach for one of three peripheral target points which appeared in a pseudo-randomized order. The peripheral target points appeared in 10 cm distance from the center point in forward (0°), forward-leftward (45° left of straight line), or forward-rightward (45° right of straight line) direction [7,10]. The subsequent movement was initiated from the peripheral point back towards the center point. Therefore, the end point of each movement was the starting point for the subsequent movement. Start and target points were displayed as light gray circles (1 cm diameter) on a black background. The cursor representing the position of the handle was displayed as white circle (0.35 cm diameter).

We defined a set of movements as six movement trials–three outward and three inward movements–in which each peripheral target point occurred exactly once and, thus, each of the possible six movements had to be performed once. To ensure the same amount of practice towards each target direction, all learning blocks were constructed as concatenation of such movement sets and all subjects experienced the same trial order.

Subjects were requested to perform each movement within 500±50 ms. Subjects were told that reaction time was not important, i.e., after appearance of the new target they could wait as long as they wanted before initiating the movement. After completion of each movement, subjects received visual feedback about movement time on the screen. If the subjects reached the target within the required time its color changed from gray to green. If they moved too slowly it became red and if moving too fast it became blue. To ensure consistent movement speed across trials, this visual feedback was provided throughout the whole experiment.

During the experiment, three different trial types were used: null field trials (NF, no perturbing forces), force field trials (FF, perturbing forces), and force channel trials (FC, error clamp trials). On null field trials, the robot’s motors were turned off and the subjects could reach without perturbing forces.

On force field trials, the robot generated velocity-dependent force fields that applied forces to the subjects’ arm via the robot handle. These force fields were used to alter the dynamic conditions of the movements such that subjects had to adapt their motor output [12]. More precisely, we used curl force fields (Fig 1C and 1D) which pushed the handle perpendicular to the direction of movement:

$$F=\left[\begin{array}{c} F_{x}\\ F_{y} \end{array}\right]=\left[\begin{array}{cc} 0 & k\\ -k & 0 \end{array}\right]\cdot \left[\begin{array}{c} \dot{x}\\ \dot{y} \end{array}\right],$$

where *F*_x_ and *F*_y_ are the robot-generated forces, *k* = 15 Ns/m is the force field viscosity, and *ẋ* and *ẏ* denote the endpoint velocity in *x*- and *y*-direction. We used two force fields of the same structure but of reversed orientation: (±*k*) a clockwise-directed force field (+*k*, Fig 1C) and a counterclockwise-directed force field (–*k*, Fig 1D). Thus, two tasks could be created: task A (force field A) and task B (force field B), where A = –B (FF_A_ = –FF_B_). The directions of the force fields were counterbalanced across all subjects. Therefore, we will refer to task A (force field A) and task B (force field B) rather than to clockwise-directed force field and counterclockwise-directed force field.

On force channel trials, the robot generated a virtual force channel (wall stiffness 6000 N/m, wall viscosity 25 Ns/m) that restricted subjects’ movements at the endpoint to a straight line toward the target point, thus, counteracting all movements perpendicular to the target direction [27]. These trials were used to measure the forces at the handle which subjects produced perpendicular to the movement direction. These forces served as indicator for predictive force field compensation. As on these trials the motor errors were clamped to zero and the force field that had to be learned was not present, these trials allowed measurement of motor adaptation with respect to feedforward adaptation without overlapping error feedback or learning mechanisms [9,27].

### Experimental design

**Fig 2:**
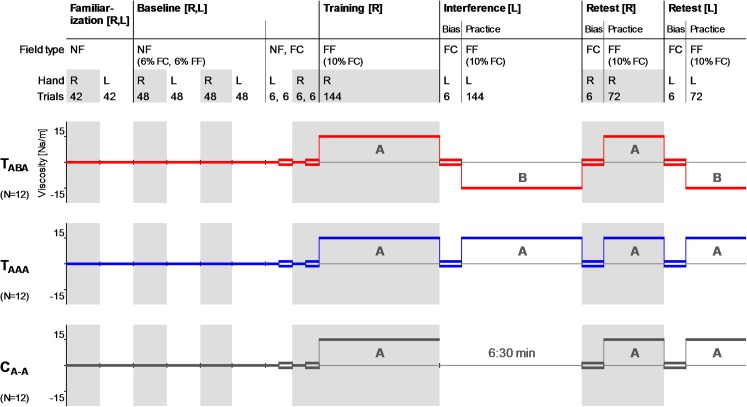
Experimental design. Practice schedules for the three subject groups T_ABA_, T_AAA_, and C_A-A_ (controls). R: right arm, L: left arm, NF: null field, FC: force channel, FF: force field. Task A and B refer to viscous curl force fields of the same structure but mirrored orientation (A = –B).

For the investigation of interlimb interference effects, we adopted the classical ABA-paradigm [18] such that subjects switched arms between the blocks. The schedule (Fig 2) comprised a familiarization/baseline block (right and left arm), a training block (right arm), an interference block (left arm), and a retest block (right and left arm). The subjects were randomly assigned to one of three groups (T_ABA_, T_AAA_, C_A-A_; 12 subjects per group; Fig 2). The test group T_ABA_ learned task A with the right arm, subsequently learned the interfering task B (= –A) with the left arm followed by a retest with the right (task A) and left (task B) arm. The test group T_AAA_ consecutively learned task A with the right and the left arm before being retested for task A with the right and left arm. The control group C_A-A_, learned task A with the right arm followed by a 6:30 min break and a retest of task A with the right and left arm. The 6:30 min break corresponded to the mean time which subjects of the test groups needed to perform the left arm interference block.

To familiarize subjects to the task conditions and to ensure consistent movement speed, the subjects initially performed familiarization and baseline trials. Consistent movement speed was important because the upcoming perturbing force field was velocity-dependent and we wanted subjects to experience a consistent amount of forces throughout the experiment. Moreover, subjects were familiarized with repeated arm switches. The familiarization block consisted of 42 null field trials per arm. The baseline block consisted of 2 cycles of 48 trials per arm, which comprised 42 null field trials as well as 3 pseudorandomly interspersed force channel trials (~6%) and 3 pseudorandomly interspersed force field catch trials. The baseline block finished with another set of null field trials followed by a set of force channel trials for each arm, respectively (1 trial per movement direction and arm). The force channel trials during the whole baseline block (2 force channel trials for each arm and each movement direction) served as baseline trials.

The force field training block (144 trials) mainly consisted of force field trials according to task A which were performed with the right arm. To assess changes in subjects’ force field predictions during training, 14 force channel trials were pseudorandomly interspersed during the training block (~10%).

The interference block was performed using the left arm and was designed similar to the previous training block. Initially, subjects performed 6 force channel trials. This allowed assessment of transfer effects in terms of a practice-dependent bias which refers to a change in the prediction of the environmental conditions when reaching with the untrained left arm caused by the previous right arm force field adaptation [9,10]. Directly afterwards, subjects performed left arm movements under force field conditions. This practice block (144 trials) mainly consisted of force field trials according to task B (T_ABA_) or task A (T_AAA_). To assess changes in subjects’ force field predictions, 14 force channel trials were pseudorandomly interspersed during the interference block (~10%). The target sequences for this interference block (left arm) was mirrored compared to the initial training block (right arm) and, thus, similar with reference to the body midline. Subjects of the C_A-A_ group had a break.

The retest block started with 6 right arm movements in force channel trials in order to assess subjects’ predictions about the dynamic conditions in terms of a practice-dependent bias. Afterwards, subjects performed 72 practice trials under force field conditions according to the initially learned task A (including ~10% force channel trials).

Finally, all subjects were also retested with their left arm for their force field prediction using another 6 force channel trials as well as another 72 practice trials under force field conditions (including ~10% force channel trials) according to task A (C_A-A_, T_AAA_) or task B (T_ABA_), respectively. Using this left arm retest block, we tested if subjects identified and predicted the correct force field direction after they repeatedly changed arms, i.e., we tested if the T_AAA_ group identified the constant presentation of force field A for both arms and if the T_ABA_ group identified the alternating presentation of force field A and B for the right and left arm, respectively. In particular, this left arm retest could show whether subjects change their preferred coordinate representation–if existent–based on experience.

Throughout the whole experiment, subjects were given short breaks of 30 s each time they had to change the reaching arm as well as after 78 trials (14 sets) in the force field training and interference blocks. During all breaks, subjects were allowed to remove their hand from the robotic handle but remained seated.

### Data analysis

#### Preprocessing

All data was processed using the custom-made software application ManipAnalysis [28]. Raw data was filtered using a fourth-order Butterworth low-pass filter with a cut-off frequency of either 6 Hz (positional data) or 10 Hz (force data). Movement velocities were numerically computed using central difference method. Data sets were segmented by defining movement start (or end) as the time-point at which movement speed exceeded (or fell under) 10% of maximal speed of that movement. Movement data was time-normalized using cubic spline interpolation.

#### Performance measurement

Given the tasks A and B, subjects had to learn to compensate forces in perpendicular direction because the force fields which had to be learned acted perpendicular to the movement direction. Thus, we concentrated on the analysis of forces (on force channel trials) and positional deviations (on force field trials) in perpendicular direction. We computed a dynamic (force field compensation factor) and a kinematic (perpendicular displacement) performance measure.

To calculate a dynamic performance measure, we considered the forces which subjects produced perpendicular to the movement direction against the virtual channel wall during force channel trials. During the baseline block, we recorded two force profiles for each arm and each movement direction. By averaging these force profiles, we assessed a baseline force profile for each arm and each movement direction, respectively. All reported dynamic data bases on baseline-subtracted force profiles. As performance measure, we computed a force field compensation factor [9] which was found by linear regression of the measured baseline-subtracted perpendicular force profile and the ideal perpendicular force profile (forces necessary to cancel the force field if it had occurred). This performance measurement using force channel trials is a good indicator for the performance of the feedforward controller as it is not confounded by error feedback and learning mechanisms. This allows analyses of the adaptation of the feedforward controller by formation of an internal model of the task [9,27]. For the C_A-A_ and T_AAA_ groups, this force field compensation factor was always calculated with respect to force field A, i.e., positive values indicate predictive force field compensation according to task A. For the T_ABA_ group, in right arm movements, the force field compensation factor was calculated with respect to force field A and in left arm movements according to force field B.

As kinematic performance measure, we considered force field trials and computed the perpendicular displacement of the endpoint (hand) from the straight line joining start and target point at maximum movement speed (PD_vmax_). The produced movement path results from the superposition of several control mechanisms (feedforward control, feedback control, impedance control) and therefore reflects net motor performance. Positive perpendicular displacement values always indicate deviations in the direction of the applied force field (C_A-A_ and T_AAA_ groups: in direction of force field A; T_ABA_ group: right arm movements in direction of force field A, left arm movements in direction to force field B).

#### Statistics

For statistical analyses of the kinematic performance measure we always considered mean values comprising a set of six movements (containing movements towards all six possible movement directions, excluding potential interspersed force channel trials). Accordingly, “initial/end performance” of a practice block refers to the set mean values at the beginning/end of that practice block.

The force channel trials, which were considered for the statistical analyses of the dynamic performance measure, were interspersed throughout a wider range of movement trials because they were pseudorandomly induced to the practice blocks. Accordingly, for the dynamic performance measure the “end performance” of a practice block refers to the mean values comprising the last six force channel trials of that practice block representing all six movement directions. In the training and interference blocks, these force channel trials appeared on trials number 90, 98, 111, 123, 132, and 141. We calculated the percentage amount of intermanual transfer from right to left arm according to

$$c_{\mathrm{transfer},\mathrm{RL}}=\left(1-\frac{\mathrm{end}\;\mathrm{performance}\;\mathrm{training}\;\mathrm{block}-\mathrm{initial}\;\mathrm{performance}\;\mathrm{interference}\;\mathrm{block}}{\mathrm{end}\;\mathrm{performance}\;\mathrm{training}\;\mathrm{block}}\right)\cdot 100\%$$

For the right and left arm, we respectively calculated the percentage amount of intermanual interference according to

$$c_{\mathrm{interference},R}=\frac{\mathrm{end}\;\mathrm{performance}\;\mathrm{training}\;\mathrm{block}-\mathrm{initial}\;\mathrm{performance}\;\mathrm{right}\;\mathrm{arm}\;\mathrm{retest}}{\mathrm{End}\;\mathrm{performance}\;\mathrm{training}\;\mathrm{block}}\cdot 100\%$$

and

$$c_{\mathrm{interference},L}=\frac{\mathrm{end}\;\mathrm{performance}\;\mathrm{interference}\;\mathrm{block}-\mathrm{initial}\;\mathrm{performance}\;\mathrm{left}\;\mathrm{arm}\;\mathrm{retest}}{\mathrm{end}\;\mathrm{performance}\;\mathrm{interference}\;\mathrm{block}}\cdot 100\%.$$

If ANOVAs revealed significant differences, Tukey’s honestly significant difference Post-hoc tests were used. When multiple analyses were conducted to address the same research question, the Holm-Bonferroni procedure (sequentially rejective Bonferroni test) [29] was used to adjust the p-values. Moreover, we performed a Pearson correlation analysis to assess how the subjects’ intermanual transfer abilities (*c*_transfer,RL_) related to the amount of intermanual interference (*c*_interference,R_). For all statistical tests, the level of significance was a priori set to p = .05. Effect sizes were determined using partial eta squared η_p_^2^ or Cohen’s d [30]. All data is presented as mean values and 95% confidence intervals. All statistical analyses were conducted using IBM SPSS software (v.22).

## Results

### Initial force field adaptation and intermanual transfer

**Fig 3:**
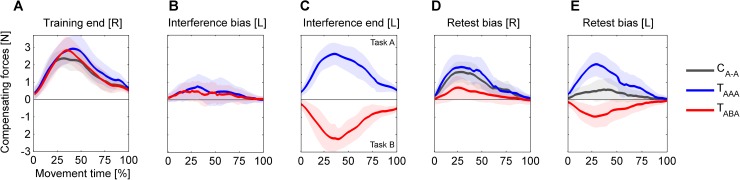
Group mean force-time curves measured during force channel trials. (**A**) Subjects learned to produce compensating forces in order to counteract the curl force fields (baseline-subtracted forces shown). At the end of the initial right arm force field training block, all subject groups adapted their force output. (**B**) On force channel trials prior to the left arm interference block, subjects showed a bias in force production according to force field task A, i.e., intermanual transfer. (**C**) At the end of the left arm interference block, the test groups T_AAA_ and T_ABA_ adapted their force output to force field task A and B, respectively. (**D**) Following the left arm interference block, the subjects’ right arm movements showed a force field prediction according to task A, which differed between groups. In particular, the T_ABA_ group’s force field prediction differed from that of control subjects indicating intermanual interference. (**E**) In the left arm retest, test subjects showed a force field prediction according to the force field which was previously learned using the left arm. Control subjects, who were first tested using their left arms, showed intermanual transfer effects comparable to that previously observed in the test groups (**B**). Values are means ± 95% confidence intervals.

**Fig 4:**
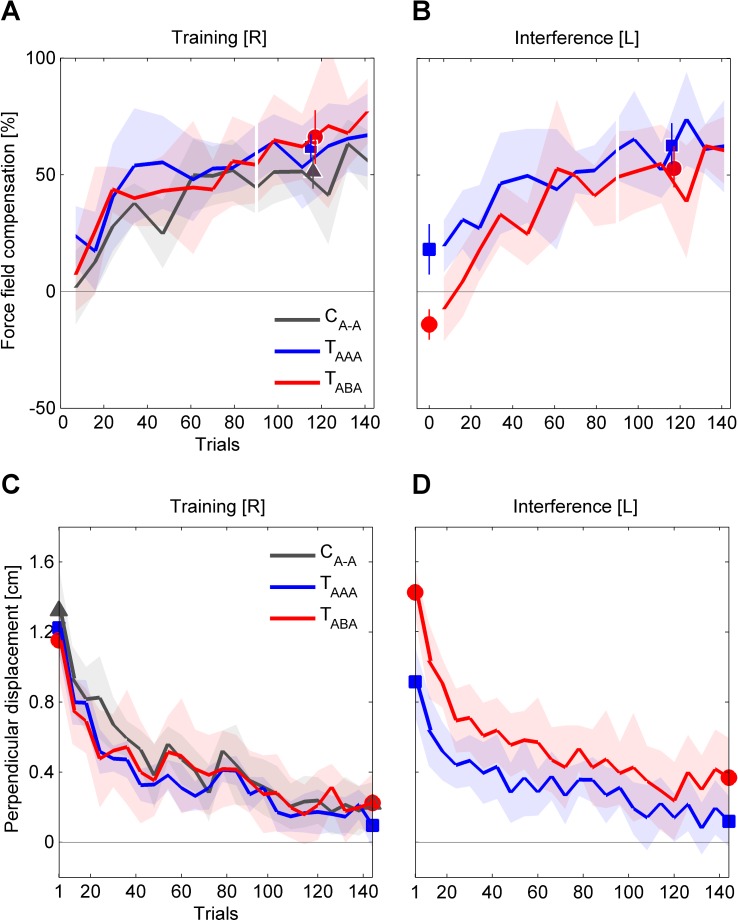
Group mean values of motor performance throughout training and interference blocks. (**A**, **B**) The performance quantified by the (baseline-subtracted) force field compensation factor was assessed on pseudorandomly interspersed force channel trials during the practice blocks and in a set of error clamp trials prior to the interference block. The last six force channel trials, represent movements towards all six movement directions (data of set mean values emphasized). (**C**, **D**) The performance quantified by the kinematic error is illustrated as progression of set mean values respectively representing all movement directions. The emphasized data points at the beginning/end of the practice blocks refer to the groups’ initial/end performances, which are considered for the statistical analyses. Values are means ± 95% confidence intervals.

On the recorded baseline catch trials, the induced force field had significant perturbing effects on the subjects’ right (p < .001) and left (p < .001) arm movements in terms of kinematic endpoint error. This effect did not differ between arms (paired samples t-test, t_(35)_ = .360, p = .721). On average, the groups’ peak movement speeds during the training block were 28.68±1.52 cm/s (C_A-A_), 29.26±2.25 cm/s (T_AAA_), and 26.96±1.66 cm/s (T_ABA_) and did not differ between groups (one-way ANOVA, factor *group* (T_ABA_, T_AAA_, C_A-A_); F_(2,33)_ = 1.6, p = .211, η_p_^2^ = .09) indicating similar velocity-dependent forces on the subjects’ hands. As expected, all subjects were able to adapt their right arm movements during the training block to the perturbing forces by producing compensating forces (Fig 3A). On average, subjects learned to compensate 51.2±7.2% (C_A-A_), 61.8±5.5% (T_AAA_), and 66.1±11.5% (T_ABA_) of the force field perturbation (end performance in training block; Fig 4A). This attained degree of adaptation at the end of the training block did not significantly differ between groups (one-way ANOVA, factor *group* (T_ABA_, T_AAA_, C_A-A_); F_(2,33)_ = 3.2, p = .060, η_p_^2^ = .16).

For the kinematic performance measure, the one-way ANOVA comparing the *groups* (T_ABA_, T_AAA_, C_A-A_) indicated no significant differences for the groups’ attained levels of performance at the end of the training block (F_(2,33)_ = .62, p = .543, η_p_^2^ = .04; Fig 4C).

At the beginning of the interference block, the test groups T_ABA_ and T_AAA_ performed a set of six force channel trials with their left arm before being exposed to the force field. Therein, we detected significant transfer in terms of a practice-dependent bias from the right to the left arm (Fig 4B). On average, T_AAA_ subjects predictively compensated 18.2±10.8% of force field A (1-sample t-test vs. 0; t_(11)_ = 3.3, p = .007). Subjects of the T_ABA_ group on average predictively compensated -14.0±6.5% of force field B (1-sample t-test vs. 0; t_(11)_ = 4.2, p = .002), which corresponds to a prediction according to force field A. In relation to the previously attained right arm performance level, the groups transferred 21.9% (T_ABA_) and 27.6% (T_AAA_) of the force field compensation to the contralateral left side as quantified by *c*_transfer,RL_.

Accordingly, the initial left arm motor performance as measured by the kinematic error (Fig 4D, first six force field trials) facing task B (T_ABA_) or task A (T_AAA_) differed significantly between groups (independent samples t-test; t_(22)_ = 4.12, p < .001, d = 1.68).

At the end of the interference block, both test groups T_ABA_ and T_AAA_ adapted their left arm movements to task B and A, respectively (Fig 4B and 4D). On average, subjects compensated 52.8±15.6% (T_ABA_) and 62.5%±14.7% (T_AAA_) of force field B and A, respectively (mean of last six force channel trials). This left arm adaptation did not differ significantly between the test groups (independent samples t-test; t_(22)_ = 1.6, p = .127, d = .64). Accordingly, the results of the kinematic error measure revealed no statistically significant difference in the attained left arm performance (independent samples t-test; t_(22)_ = 1.76, p = .092, d = .72).

### Intermanual interference

The main goal of this study was to consider intermanual interference effects in an ABA-paradigm. For this purpose, we used two different approaches: force channel trials to detect subjects’ force field prediction with respect to task A and force field trials to detect subjects’ net motor performance facing task A.

**Fig 5:**
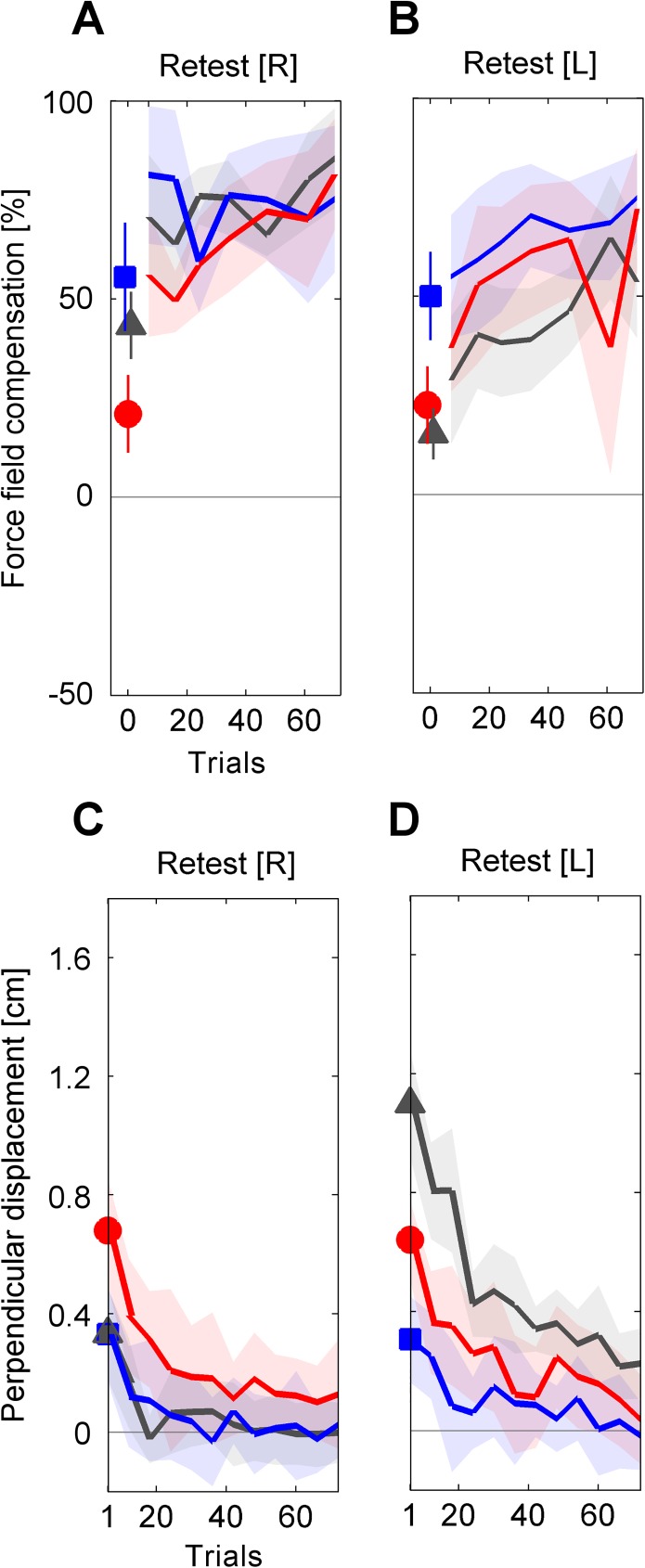
Group mean values of motor performance throughout retest blocks. (**A**, **B**) The performance quantified by the (baseline-subtracted) force field compensation factor was assessed in a set of error clamp trials prior to the retest blocks and on pseudorandomly interspersed force channel trials during the retest blocks. (**C**-**D**) The performance quantified by the kinematic error is illustrated as progression of set mean values respectively representing all movement directions. The emphasized data points at the beginning of the retest blocks refer to the groups’ initial performances, which are considered for the statistical analyses. Values are means ± 95% confidence intervals.

**Fig 6:**
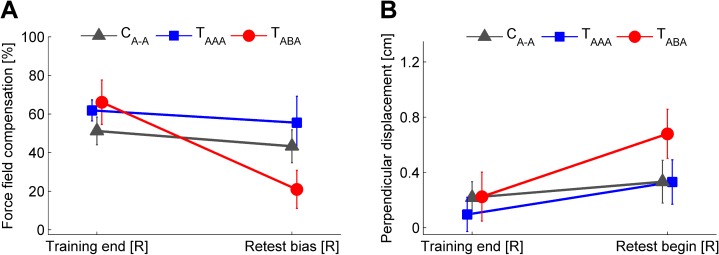
Motor performance after right arm adaptation and at right arm retest. Comparison of group mean right arm motor performance at the end of the initial training block and at the beginning of the retest block immediately after the left arm interference block considering force field compensation (**A**, baseline-subtracted) and the kinematic error measure (**B**). The impaired retention for the T_ABA_ group compared to both other groups indicates intermanual interference. Values are means ± 95% confidence intervals.

In the force channel trials at the beginning of the retest block prior to the force field exposure (retest bias [R], Fig 5A), subjects showed a decreased force field compensation compared to the end of the initial training block (Fig 6A). On average, this force field compensation decreased by 68.3% (T_ABA_), 10.2% (T_AAA_), and 15.5% (C_A-A_) compared to the initially attained performance level as quantified by *c*_interference,R_. The 2×3 ANOVA (factors: *time* (training end [R], retest bias [R]), *group* (T_ABA_, T_AAA_, C_A-A_)) revealed a significant effect of *time* (F_(1,33)_ = 29.4, p < .001, η_p_^2^ = .47) and a significant interaction of *time* and *group* (F_(2,33)_ = 12.04, p < .001, η_p_^2^ = .42). For further consideration of this significant interaction of *time* and *group*, we performed Holm-Bonferroni corrected pairwise post hoc 2×2 ANOVAs (factors: *time* (training end [R], retest bias [R]), *group*) which indicated significant interactions between *time* and *group* for T_ABA_ and T_AAA_ (F_(1,22)_ = 19.9, p < .001, η_p_^2^ = .48) as well as for T_ABA_ and C_A-A_ (F_(1,22)_ = 17.0, p < .001, η_p_^2^ = .44) but not for T_AAA_ and C_A-A_ (F_(1,22)_ = 2.4, p = .862, η_p_^2^ = .001). Note, that the force field prediction of group T_ABA_ was (like in the other groups) directed according to task A (which was initially learned with the right arm) rather than to task B (which was learned immediately before using the left arm). However, this prediction was weaker compared to both other groups indicating intermanual interference for group T_ABA_. For the T_ABA_ group we found a correlation between the amount of intermanual transfer *c*_transfer,RL_ and the amount of intermanual interference *c*_interferenceR_, which however was not statistically significant (r = .522, p = .081).

We obtained similar findings when considering the kinematic performance measure. The motor performance at the end of the right arm training block (Fig 4C) and the initial set of force field trials in the retest block (Fig 5C) showed a significant effect of *time* (2×3 ANOVA, factors: *time* (training end [R], retest begin [R]), *group* (T_ABA_, T_AAA_, C_A-A_); F_(1,33)_ = 35.1, p < .001, η_p_^2^ = .52; Fig 6B) and a significant interaction of *time* and *group* (F_(2,33)_ = 7.0, p = .003, η_p_^2^ = .30). Holm-Bonferroni corrected pairwise post hoc 2×2 ANOVAs (factors: *time* (training end [R], retest begin [R]), *group*) revealed significant interactions between *time* and *group* for T_ABA_ and T_AAA_ (F_(1,22)_ = 5.9, p = .048, η_p_^2^ = .21) as well as for T_ABA_ and C_A-A_ (F_(1,22)_ = 13.1, p = .003, η_p_^2^ = .34) but not for T_AAA_ and C_A-A_ (F_(1,22)_ = 1.25, p = .275, η_p_^2^ = .05). Thus, the retention of group T_ABA_ significantly differed from both other groups indicating an intermanual interference effect of task B onto retest of task A. However, despite consecutive learning of task A, the T_AAA_ group did not show an increased retest performance of task A compared to the control group C_A-A_.

### Sequential right-left arm motor learning

After another brief right arm learning period of task A in the retest block (Fig 5A and 5C), subjects were again tested for their left arm motor performance (Fig 5B and 5D) to test if they identified and predicted the correct force field direction after they repeatedly changed their reaching arms.

**Fig 7:**
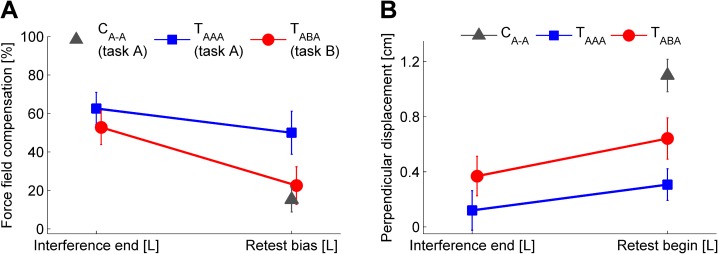
Motor performance after left arm interference block and at left arm retest. Comparison of group mean left arm motor performance at the end of the interference block and at the beginning of the retest block considering force field compensation (**A**, baseline-subtracted) and the kinematic error measure (**B**). Note that groups C_A-A_ and T_AAA_ were exposed to force field A, whereas group T_ABA_ was exposed to force field B. Accordingly, the force field compensation factor (**A**) was calculated with respect to either force field A (C_A-A_, T_AAA_) or force field B (T_ABA_). Values are means ± 95% confidence intervals.

First, we considered subjects’ predictions about the force field conditions prior to the force field exposure. On average, the predicted force field at left arm retest was decreased by 57.2% (T_ABA_, task B) and 20.1% (T_AAA_, task A) in relation to the previously gained left arm adaptation level as quantified by *c*_interference,L_. Accordingly, the 2×2 ANOVA (factors: *time* (interference end [L], retest bias [L]), *group* (T_ABA_, T_AAA_), Fig 7A) showed a significant effect of *time* (F_(1,22)_ = 34.7, p < .001, η_p_^2^ = .61) and a significant interaction of *time* and *group* (F_(1,22)_ = 5.9, p = .024, η_p_^2^ = .21). Similarly, comparing the force field prediction at the beginning of the left arm retest block (prior to the force field exposure) across all three groups revealed a significant group difference (one-way ANOVA, factor: *group* (T_ABA_, T_AAA_, C_A-A_); F_(2,33)_ = 14.7, p < .001, η_p_^2^ = .47). Tukey post hoc tests indicated significant differences between groups T_AAA_ and T_ABA_ (p = .001) as well as between T_AAA_ and C_A-A_ (p < .001). However, it is important to note, that groups T_ABA_ and C_A-A_ did not show differences in the amount of force field prediction (p = .534) but their predictions were towards different directions, i.e., according to task B for group T_ABA_ and according to task A for group C_A-A_. That is, group T_ABA_ changed their prediction about the left arm task (from task A to task B) compared to their initial left arm prediction prior to the interference block (Fig 3B vs. 3E or Figs 4B vs. 5B), thus, accounting for the alternating force fields A and B for the right and left arm, respectively.

Analyses of the motor performance quantified by the kinematic error measure (2×2 ANOVA, factors: *time* (interference end [L], retest begin [L]), *group* (T_ABA_, T_AAA_); Fig 7B) indicated a significant effect of *time* (F_(1,22)_ = 22.0, p < .001., η_p_^2^ = .82) but no significant interaction of *time* and *group* (F_(1,22)_ = 1.3, p = .267., η_p_^2^ = .06). Comparison of all three groups’ retest performances facing task A (T_AAA_, C_A-A_) or task B (T_ABA_) revealed significant differences between groups (one-way ANOVA, factor: *group* (T_ABA_, T_AAA_, C_A-A_); F_(2,33)_ = 26.4, p < .001, η_p_^2^ = .62). Tukey post hoc comparisons showed significant differences between each two groups, respectively (p≤.012). Thus, alternating right-left arm learning of different tasks A and B significantly influenced the prediction about the task as well as the motor performance with respect to these tasks.

## Discussion

The main finding of our study is that consecutive learning of two unimanual tasks using different arms leads to interference of motor memory. In particular, we found that this intermanual interference occurred when the interfering task was mirror-symmetric with respect to the body midline (same task structure but mirrored orientation) whereas such interference did not occur when the interfering task was equal with respect to an extrinsic coordinate frame. This indicates a preferred internal task representation in an extrinsic reference frame. However, consecutive learning of the same task (extrinsically consistent) with both arms did not lead to a facilitation of motor performance which is presumably attributed to ceiling effects of learning.

### Consecutive learning of opposing tasks using different arms causes intermanual interference

As hypothesized, we detected transfer of motor adaptation across arms with respect to an extrinsic coordinate frame [7,10,11,31]. Thus, intermanual transfer of task A resulted in a motor output according to task A on the contralateral side rather than according to the mirrored task B = -A (Fig 3B). Consequently, the T_ABA_-group started the interference block with a force field prediction which was opposed to the dynamics of the upcoming task B, whereas the T_AAA_-group started this block with a matching force field prediction with respect to the upcoming task A (Fig 4B). Accordingly, we hypothesized that consecutive right-left arm learning in an ABA-schedule leads to motor memory interference because task B does not match subjects’ preferred task transfer pattern. Our results support this hypothesis as the group that learned in the ABA-schedule showed significantly impaired task A motor performance at retest compared to control subjects or subjects learning in the AAA-schedule (Figs 3D and 6). Motor memory interference can be of anterograde or retrograde fashion. In anterograde interference, learning of a task impairs subsequent learning of another task. Retrograde interference refers to an interference of task learning onto a previously formed memory of another motor task [19–21]. We did not aim to distinguish anterograde from retrograde interference effects but rather to discover intermanual interference effects in general. Previous studies using a classical ABA-paradigm, showed that both anterograde and retrograde interference affect retest performance of task A [19,20]. Presumably, our observed intermanual interference effects are also of both anterograde and retrograde source.

Our behavioral findings on intermanual interference in an ABA-paradigm suggest a preferred internal representation of motor actions in extrinsic coordinates. It is noteworthy that task A and B were of similar structure but were only mirrored about the midsagittal plane, i.e., equivalent with respect to an intrinsic joint-based reference frame (A = -B; Fig 1C and 1D). If motor memories were primarily represented in an intrinsic reference frame, subjects should be able to utilize the experience of left arm learning (task B) for an improvement rather than a decrease in the right arm motor performance (task A).

This finding also holds for the left arm retest of task B. Subjects learning in the ABA-schedule started the left arm retest with a force field prediction that was (oppositely directed but) not stronger than in control subjects who did not perform any left arm force field training before (Figs 3E and 7A). Thereby, the control group’s feedforward model could only rely on transferred motor learning from the right to the left arm. The feedforward model of T_ABA_ subjects could utilize motor memory obtained by both intermanual transfer of motor learning and prior left arm motor learning. Indeed, subjects of the T_ABA_ group reversed the direction of their motor prediction (compared to the initial transfer bias from the right to the left arm) towards task B but their feedforward model of task B did not differ in magnitude from the control group’s feedforward model of task A (Figs 5B and 7A). In other words: even when subjects were forced to learn the force field task on the contralateral left side in an intrinsically equivalent fashion, they were not able to utilize this experience to quantitatively improve their motor performance compared to controls. Rather, the more extensive training in the ABA-schedule was needed to recognize the alternating task conditions and to reverse the default prediction, which based on the preferred extrinsic coordinate representation of the task.

### Arm switch as contextual cue to learn competing tasks

Another viewpoint would be to consider the arm switch in the ABA-schedule as contextual cue for learning two competing tasks. As initially mentioned, several previous studies demonstrated motor memory interference when two different tasks were learned using the same arm [17,18,21–25,32]. In some cases, this interference was shown to be reduced when associating the competing tasks with visual or proprioceptive context cues [33–37]. In most of these investigations, the task conditions randomly varied on a trial-by-trial basis. Thus, the context switch was performed more often compared to our schedule. In our experiment, subjects learning in the ABA-schedule complied with the alternating pattern of the force field direction when switching the arms for the third time (from right arm retest to left arm retest; Figs 3E and 5B). As mentioned above, these subjects reversed their force field prediction but the amount of force field compensation did not differ from controls. Thus, for those subjects, the more extensive training and switching arms was necessary to overcome the preferred internal task representation (in extrinsic reference frame). We can only speculate how further sequential learning of the two opposing tasks would evolve if subjects changed their reaching arms more often. But based on our results and on former investigations on proprioceptive context cues (e.g., [35]), it is likely that subjects could learn both tasks using the arm switch as contextual cue and therewith reduce interference effects. Such use of each arm as context switch has previously been reported for concurrent adaptation to visual distortions [38,39].

### Learning the same task with both arms did not facilitate motor performance

One might argue that an internal representation of our task in an extrinsic reference is reasonable because subjects associate the altered dynamics with the robotic handle, i.e., an external object. Accordingly, there is no reason for the subjects to assume that the robot behaves differently (e.g., mirrored) when grasping it with the contralateral hand. In this case, learning to handle the robotic handle should result in an elaboration of an internal model its dynamics irrespective of the arm used. However, in contrast to our hypothesis, subjects who learned in the AAA-schedule did not show a significantly increased task A retest performance compared to control subjects (Figs 3D and 5A). Thus, they were not able to benefit from more extensive learning of the same task. Possibly, this is attributed to a ceiling effect of learning [40]. Subjects learning in the AAA-schedule already reached a comparably high level of performance at the end of the initial learning block (Fig 4A and 4C). Maybe, this could not be further improved by contralateral learning. It is possible that such a facilitation of motor performance would be detectable when a more complex task is learned or when the retest follows a longer rest period. Further studies should consider more complex motor tasks in order to gain more practical insights for the design of effective training schedules in rehabilitation and sports settings (e.g., bilateral training schedules [41]).

### Mechanisms underlying motor memory interference

To our best knowledge, this is the first study considering intermanual interference in motor learning of tasks sharing the same structure. Considering the control group, approximately 15% of the subjects’ decrement in right arm motor performance is attributed to temporal factors, e.g., forgetting or loss of internal states [40,42]. Learning in the ABA-schedule, yielded a more pronounced performance decrement (approximately 68%) which could be explained by temporal factors plus motor memory interference factors (Fig 6). Thus, our behavioral findings considering the right arm retest performance could be explained by a superposition of competing motor memories of task A and task B [43]. Recent investigations as well as our results indicate that subjects transfer approximately 10–30% of learning to the contralateral side which alters the forward model of the task [9–11]. Combining the (negative) contralateral transfer effect of task B onto retest of task A with the decrement due to temporal factors qualitatively accounts for the distinct reduction of task A motor memory at retest in the ABA-schedule. Clearly, such a superposition of competing motor memories is oversimplified because it would also predict complete unlearning of task A if task B is learned on the contralateral arm for a longer period. Such complete unlearning of motor memory is rather unlikely.

Moreover, such a simple superposition should only be possible, when the competing tasks A and B are of similar structure and are entirely processed by the same neural networks. Lauber and colleagues [44] also found intermanual interference effects considering competing tasks which were of different structure (ballistic task and accuracy task). Therefore, it remains elusive in which manner the task structure and the involved neural networks of competing tasks relate to their memory interference. Our behavioral results on intermanual transfer and intermanual interference support the idea that motor tasks which share the same structure but are executed using different effectors at least partly involve similar neural networks. Similarly, bimanual movements were shown to require substantive interhemispheric interactions [45–47]. If the amount of intermanual transfer depends on the strength of interhemispheric interactions or the amount of shared neural networks for motor control and learning of the left and the right arm, the amount of intermanual transfer and the amount of intermanual interference should correlate. Our results indicate such a correlation which, however, was not statistical significant. Thus, such a dependency should be thoroughly investigated in future studies. As we did not record or modulate subjects’ neural activity, we cannot state specific brain regions which might be involved. However, the behavioral results support the idea of a vital interhemispheric connectivity in terms of a bilateral access of unilaterally formed motor memory (bilateral access model) or a bilateral neural activity during unilateral motor control and learning (cross-activation model [2]. To identify specific brain regions underlying control and learning of arm movements, further neurophysiological studies are necessary.

## Conclusion

This study demonstrates clear intermanual interference effects when subjects consecutively adapt their arm movements to two opposing force field tasks using the right and the left arm, respectively. The force field tasks where of the same structure but only their directions were mirrored about the midsagittal plane. Contrary, intermanual interference was absent when subjects consecutively learned the same force field with both arms. Thus, our results comply with the assumption of a preferred internal task representation in a Cartesian-based extrinsic coordinate frame rather than in a joint-based intrinsic coordinate frame. Moreover, our behavioral results support the assumption that motor control and learning of right and left arm movements partly involve similar neural networks. Altogether, these findings contribute to an enhanced understanding of the mechanisms underlying motor control and learning of arm movements. Further investigations should consider the actually involved neural networks underlying intermanual transfer using functional imaging techniques. Furthermore, in order to gain more practically relevant insights for the design of bilateral practice schedules, investigations considering more complex movements are necessary.

## Supporting information

S1 File Force field compensation data for the three subject groups. (CSV)

S2 File Perpendicular displacement data [m] for the three subject groups. (CSV)
